# The Cost-Effectiveness of a Personalised Early Warning Decision Support System (The COPDPredict™ System) to Predict and Prevent Acute Exacerbations of Chronic Obstructive Pulmonary Disease

**DOI:** 10.2147/COPD.S486309

**Published:** 2025-05-25

**Authors:** James A Hall, Alice M Turner, Eleni Gkini, Rajnikant Mehta, Monica Spiteri, Neil Patel, Sue Jowett

**Affiliations:** 1 Health Economics Unit, Department of Applied Health Sciences, University of Birmingham, Birmingham, UK; 2 Department of Applied Health Sciences, University of Birmingham, Birmingham, UK; 3 Birmingham Clinical Trials Unit, Department of Applied Health Sciences, University of Birmingham, Birmingham, UK; 4 Centre for Evaluation and Methods, Wolfson Institute of Population Health, Queen Mary University of London, London, UK; 5 NEPESMO Ltd, London, UK

**Keywords:** chronic obstructive pulmonary disease, respiratory medicine, self-management, digital health, cost-effectiveness analysis, decision modelling

## Abstract

**Purpose:**

Chronic obstructive pulmonary disease (COPD) is a respiratory disease associated with significant morbidity, mortality, and healthcare burden. Many COPD patients are frequent exacerbators, which has a significant impact on patient prognosis. Prompt exacerbation management using a digital tool, COPDPredict™ may support COPD patients in identifying exacerbations earlier to reduce hospital admissions.

**Methods:**

Trial-based cost-utility and cost-effectiveness analyses from the UK National Health Service perspective compared the cost-effectiveness of COPDPredict™ with usual care for a COPD GOLD stage B and D cohort. A model-based analysis was also performed by extrapolating data from the trial to obtain the-cost-utility over a 5-year time horizon. The de-novo model was constructed using GOLD stages A–D as the health states.

**Results:**

The imputed trial-based analysis showed that at a willingness to pay £20,000 per quality-adjusted life-year (QALY), COPDPredict™ was 65% likely cost-effective in COPD B and D patients over 6-months with an incremental cost-effectiveness ratio (ICER) of £11,669/QALY (incremental cost +£238.16 (106.42), Incremental QALY +0.02 (0.012)). The results were robust to complete case analyses over 6- and 12-months. A similar ICER (£11,862/QALY) was obtained when performing model-based analysis over 5-years. Cost-effectiveness was sensitive to long-term effectiveness, cost parameters and alternative model structure, with expected value of information analyses suggesting a significant benefit from future research targeting the long-term effectiveness of the intervention.

**Conclusion:**

COPDPredict™ is potentially cost-effective for COPD B and D patients. However, the small samples sizes upon which the results were obtained warrant further investigation.

## Introduction

Chronic obstructive pulmonary disease (COPD) is a progressive lung disease affecting tens of millions of adults globally.^1^ The disease is accompanied by recurring acute exacerbations (AECOPD), that lead to clinical deterioration, and when severe, cause around 130,000 hospital admissions annually in the UK.^2^ The UK has one of the highest mortality rates from lung disease in Europe,^3^ with nearly 40% of patients dying or being readmitted to hospital within 90 days of an exacerbation of COPD.^4^ A 2022 study estimated that the healthcare costs of COPD in England was around £2bn and estimated to continue to increase^5^ to £2.32bn by 2030.^6^

National Institute for Health and Care Excellence (NICE) treatment guidance for COPD^7^ includes a supported tailored self-management plan (SSMP) for patients to identify and treat AECOPD, typically by taking rescue medications (antibiotics and steroids). Various treatments are recommended by NICE to reduce exacerbations and improve care; pharmacological treatments such as azithromycin, inhaled bronchodilators, and inhaled corticosteroids; and non-pharmacological treatments such as oxygen therapy, long-term non-invasive ventilation, or pulmonary rehabilitation.^7^

AECOPD often involves shifts in key biological markers, such as blood C-reactive protein (CRP), physiological indicators (eg lung function), and worsening symptoms. Integrating these data into patient assessments can enhance the accuracy of diagnosing, determining causes, or assessing severity. While some studies have utilised CRP measurement for treatment decisions, none have incorporated multiple measures or personalised guidance.^8^

Modern digital technologies enable regular monitoring of physiology, pathology, and symptoms for personalised early AECOPD warnings. This empowers patients, with or without healthcare support, to potentially avoid hospitalisation. Spirometry measurements, connected to symptom reporting via a Bluetooth-enabled app, can be performed at home, and point-of-care CRP tests can offer real-time biomarker data. Based on these principles, the COPDPredict™ system, an approved medical device, has shown in preliminary data it may reduce admissions in COPD patients. The Predict & Prevent Phase III two-arm, multi-centre, open-label, parallel-group, individually randomised clinical trial of the device was undertaken to determine whether the system could be adopted in UK practice.^8^

Recent clinical evidence suggested that the COPDPredict™ system is associated with lower hospital admissions and improved quality of life.^9^ Adoption decisions regarding new healthcare interventions in England and Wales require recommendations from NICE guidance based on clinical and cost-effectiveness evidence, with economic evaluations from an NHS/Personal Social Services (PSS) perspective using cost-utility analysis (cost-per-quality-adjusted-life-year (QALY)).^10^ The NHS/PSS perspective is preferred for economic evaluations in the UK, as interventions are funded directly by the NHS, which, in turn, is centrally funded by general taxation and national insurance via the Department of Health and Social Care. Economic evaluation evaluates cost-effectiveness by calculating the estimated incremental costs and effects arising from the introduction of a new technology compared with current care. Accordingly, using data from the Predict and Prevent trial, we present an economic evaluation of both cost-per-QALY gained and cost-per-disease-specific measure using cost-per-additional exacerbation prevented. Where the costs and effects arising from the introduction of a new technology are expected to last beyond the time horizon of the trial, it is recommended that decision modelling be undertaken to estimate the long-term cost-effectiveness of the intervention. With this in mind, we also present the modelled cost-effectiveness of the COPDPredict™ system over 5 years from the NHS/PSS perspective.

## Methods

### Overview

Full details of the Predict and Prevent trial methods are presented in the trial protocol^8^ and clinical effectiveness paper.^9^ In brief, a Phase III, multi-centre, individually randomised trial with 12-month follow-up investigated the efficacy of a personalised early warning decision support system (COPDPredict™) in predicting and preventing acute COPD exacerbations. The eligibility criteria were a clinical diagnosis of COPD confirmed through post-bronchodilator spirometry, and a history of one or more AECOPD episodes within any 12-month period in the past 2 years, or one or more hospital admissions for AECOPD within the preceding 2 years. Participants were excluded if they had a life expectancy under 12 months or had comorbidities that precluded patients from using the intervention. Due to recruitment difficulties, the final sample size was 90 patients, with 45 patients in each arm, and a follow-up period of 6–12 months. Therefore, patients with shorter follow-up periods were also included in the analysis to increase sample size.

Accordingly, the base case within-trial economic evaluation was performed over 6-months follow-up using imputation according to the intention-to-treat principle for participants providing economic data at least one follow-up point. All costs are reported in 2021 British pounds (£). The analysis was undertaken from the UK NHS/PSS perspective as per NICE guidance for economic evaluations.^10^ The cost-utility analysis calculated the cost-per-additional QALY gained, as well as a cost-effectiveness analysis of cost-per-hospitalisation prevented. STATA 17 (StataCorp. 2021. *Stata Statistical Software: Release 17*. College Station, TX: StataCorp LLC). and Excel (Microsoft Corporation. 2022. Microsoft® Excel® (Version 2202 Build 16.0.14931.20806) 64-bit) was used for analysis.

### Trial-Based Economic Evaluation

#### Health Care Resource Use and Costs

Resource use data were collected via questionnaires at 3-, 6-, 9-, and 12-months post-randomisation, respectively. The questionnaires documented details on AECOPD-related hospitalisations, respiratory clinic visits, A&E attendance, investigations, primary care visits, and medication use. Resource use was costed using standard unit costs (see Appendix 1), including NHS reference costs,^11^ NHS electronic drug tariff^12^ for medications, and Unit Costs for Health and Social Care.^13^

A detailed cost analysis involving communication with intervention developers, study members, and independent stakeholders determined NHS costs for the COPDPredict™ intervention (Table 1). It was assumed that most patients (70%) would receive the App within a hospital setting with the annual licence paid by the health system. The intervention would require clinical support for ongoing monitoring of the clinical dashboard (with a training cost in the first year). CRP testing and home spirometry would be ongoing, with initial costs of purchasing testing equipment in the first year.

**Table 1:** Annual Costs Associated with COPDPredict™

Licencing and IT	
Implementation of IT	£5.00
Tech support/maintenance/data storage	£40.00
**Licence fee for COPDPredict (inc Implementation & support)**	**£120.00**
% of patients requiring tablet (estimated number of patients required a tablet to use the App)	15.00%
Mean cost of Tablet	£100.00
**Weighted cost of tablet per patient**	**£15.00**
**Prescription & set up costs**	
Community prescribing (40% band 4 clinical administrator 25 mins; 40% band 6 nurse 25 mins; 20% GP appointment 18 mins)	£53.60
Hospital prescribing (25 mins band 6 nurse – within CQUIN discharge bundle)	£31
Mix of prescription (Hospital) (estimated set up to take place mostly within hospital setting)	70%
**Prescription & set up cost**	**£37.96**
**Clinical support^a^**	
Workforce Clinical dashboard support (costs of monitoring assumed to be 2 FTE band 4 administrators and 0.5 FTE band 6 nurse per 1000 patients)	£65.78
**Costs of clinical support**	**£65.78**
**Cost of App and algorithm in first year**	**£238.73**
**Cost of App and algorithm in subsequent years (minus set-up costs)**	**£200.78**
**Blood CRP testing**	
POC IVD Analyser for Blood CRP, per patient (one-off costs £1600 per 1000 patients, lasts 5 years)	£1.60
Annual cost of assay cartridges per patient (one CRP test every 2 months)	£30.00
**Cost of Blood CRP testing, first year**	**£31.60**
**Annual cost of Blood CRP testing, subsequent years**	**£30.00**
**Other costs**	
Spirometer - Smart BT Unit cost (one-off cost, first year)	£90.00
Training for healthcare professionals in first year (1 hour training for band 6 nurse and 1 hour for band 4 clinical administrator, one-off cost, first year)	£2.24
**Cost of COPD-SPOC in first year**	**£362.57**
**Cost of COPD-SPOC in subsequent years**	**£231.23**

**Notes**: ^a^Home visits related to delivery of the intervention are costed within the healthcare resource usage, results shown below in Table 2.

**Table 2:** Unadjusted Imputed Health Outcomes Over 6 months for Patients at Baseline, 3 and 6 Months. Costs at 6 Months. Values are Mean (SE) Scores Unless Stated

**Mean EQ-5D^a^**					**6-Month QALYs^b^**
**Strategy**	**N**	**Baseline**	**3 Months**	**6 Months**	
COPD Predict	39	0.529 (0.034)	0.537 (0.040)	0.587 (0.037)	0.274 (0.016)
Usual care	41	0.487 (0.039)	0.463 (0.041)	0.490 (0.436)	0.238 (0.018)
Incremental difference		+0.042 (0.052)	+0.073*(0.057)	+0.097 (0.058)**	+0.036 (0.024)
**Cost Type**	***Mean Cost (£), (SE)***				
	**Intervention, n=39**		**Usual Care, n=41**		**Mean Difference**
NHS costs					
Intervention cost	£181.29		0		+£181.29
Home visit costs	£125.43 (25.30)		0		+£125.43 (24.67)
Primary care costs	£73.41 (25.13)		£51.16 (11.09)		+£22.25 (27.01)
A&E admission cost	£34.37 (19.46)		£34.93 (15.97)		-£0.55 (25.07)
Pharmaceutical costs	£212.53 (21.54)		£256.33 (17.03)		-£43.80 (27.31)
Dom NIV / Pulmonary rehab	£170.40 (63.80)		£133.89 (44.09)		+£36.51 (76.91)
Hospitalisation costs	£262.26 (103.58)		£380.38 (169.78)		-£118.12 (208.13)
**Total NHS resource use**	**£1.059.69 (155.08)**		**£856.69 (200.63)**		**+£203.00 (255.36)**
Private healthcare costs^c^	£6.32 (5.38)		£28.97 (20.60)		−£22.65 (17.59)
**Total healthcare resource use**	**£1,066.01 (176.44)**		**£885.66 (207.42)**		**+£180.35**

**Notes**: *Statistically significant at p<0.10. **Statistically significant at p<0.05. ^a^Unadjusted EQ-5D scores with chained imputation controlling for disease severity, gender, age, site and primary outcome. 10 patients returned zero questionnaires post baseline and were excluded from the imputation. ^b^QALYs unadjusted (adjusted estimates shown in Table 6). ^c^Private healthcare costs include costs of privately obtained healthcare as well as patient incurred costs of purchased medical devices.

#### Patient Costs and Work-Related Absence

Data on direct patient medical costs related to purchasing medical devices or paying for private healthcare were obtained from the questionnaires. Productivity costs were calculated using the human capital approach by multiplying self-reported absence by the respondent’s wage based on UK occupational classification coding and annual earnings for each job type, and assuming that the value of lost work is equivalent to compensation.^14^ Due to the small number of responses and the risk of spurious correlation these outcomes were not included in the cost-effectiveness analysis. Patient direct private healthcare costs are shown in Table 2, and outcomes related to work-related absence are reported in Appendix 2.

#### Outcomes

The EQ-5D-5L instrument generates a utility score based on health-related quality-of-life preferences. EQ-5D-5L scores were obtained using questionnaires at baseline and 3-, 6-, 9- and 12-months post-randomisation. EQ-5D-5L responses were converted into multi-attribute utility scores using an approved “cross-walk” to the 3 L instrument for the UK.^15^ QALYs were calculated using the area under the curve method, assuming linear interpolation between time periods. Deceased patients were excluded from the analysis as neither comparator was expected to impact mortality, and their inclusion may bias estimates due to small sample sizes as an early death would result in a QALY very close to zero. For the cost-effectiveness analysis, self-reported hospitalisations for either infective or non-infective COPD exacerbations were used as the outcome measure.

#### Statistical Analyses

Imputed mean healthcare costs and health-related quality of life (measured by EQ-5D-5L and QALYs) were presented for each intervention during 6-month follow-up. In the base-case cost-utility and cost-effectiveness analyses, missing cost and outcome data were analysed using intention-to-treat principles. Chained multiple imputation was utilised, using the arithmetic mean of 50 estimates for each missing value, and considering predictors of missingness, such as previous or subsequent observations of the same variable, age, gender, site, and COPD stage. Hospital admission for exacerbation was part of the imputation model for costs. Disaggregation of all mean costs related to the intervention and usual care, healthcare, patient-incurred costs, and time-off work were presented for the base case. Due to substantial missing data, a 12-month imputed analysis was presented as a speculative sensitivity analysis. Complete case analyses were shown for the 6-month and 12-month periods, using data from patients who completed all healthcare and EQ-5D questionnaires for those time points. The costs and QALYs were not discounted.

Incremental cost-effectiveness ratios (ICERs) were computed by dividing the adjusted difference in mean costs by the adjusted difference in mean QALYs for the cost-utility analysis and the adjusted difference in hospitalisations for the cost-effectiveness analysis, using non-parametric bootstrapping with 5,000 replications. Adjustment was made through seemingly unrelated regression of incremental costs and effects, incorporating baseline variables (age, gender, site, and COPD stage) along with the baseline EQ-5D for QALYs. The probability of cost-effectiveness was evaluated using the NICE threshold, reflecting the UK health system’s willingness to pay for an additional QALY, a threshold which lies between £20,000 ($25,000 or €23,400) and £30,000 ($37,300 or €35,100) per additional QALY.

Base case results were graphically depicted in cost-effectiveness planes and acceptability curves (CEACs), displaying the distribution of incremental costs and QALYs, and estimating cost-effectiveness across various willingness-to-pay (WTP) thresholds for an additional QALY. Deterministic sensitivity analysis assessed robustness by undertaking a complete case analysis as well as 12-month imputed and complete case analyses.

### Model-Based Analysis

#### Overview

A cost-utility analysis extrapolating beyond the trial was performed by comparing COPDPredict™ with usual care for management of patients with COPD stage D. A Markov cohort model with a 1-month time cycle was constructed to estimate QALYs and costs from the NHS/PSS perspective. A 5-year time horizon was chosen, reflecting the duration experts felt the technology would benefit patients. Discounting was applied at 3.5% for both the costs and outcomes.

#### Model Population

As nearly all participants in the trial had COPD stage D (more COPD symptoms as well as ≥ 2 AECOPD or ≥ 1 hospitalised exacerbation) (87.8% stage D and 12.2% stage B), only this patient group had reliable parameter estimates; therefore, the base-case model was run exclusively for patients in COPD GOLD stage D.

#### Model Structure and Transition Probabilities

In the first year, the costs and QALYs for both comparators were derived from adjusted bootstrapped analyses of the trial data (Appendix 3 and Table S4). From year 1 onwards, patients began in health states that reflected the GOLD stage achieved in the trial at 12-months (see Table 3). COPDPredict™ had fewer patients in Stage D compared with usual care, resulting in a beneficial effect throughout the model.

**Table 3:** Proportions in Each Health State at 12 months with PSA Beta Distribution Parameters

COPD Stage^b^	Usual Care Proportion (Alpha, Beta)	COPDPredict™ Proportion (Alpha, Beta)
**A**	0 (0.05^a^, 23)	0 (0.05^a^, 17)
**B**	0.130 (3, 20.05)	0.176 (3, 14.05)
**C**	0 (0.05^a^, 23)	0.059 (1, 16.05)
**D**	0.870 (20, 3.05)	0.765 (13, 4.05)

**Notes**: ^a^Alpha parameters limited to 0.05 were selected for stages with zero occupancy in the trial due to small sample sizes (to limit the impact of adding an integer to the overall probabilities sampled by the alpha within the PSA). ^b^GOLD A (low risk, fewer symptoms), B (low risk, more symptoms), C (high risk, fewer symptoms), and D (high risk, more symptoms). Fewer symptoms reflects COPD Assessment Test (CAT) score <18, more symptoms CAT score ≥18. High risk reflects patients with ≥2 exacerbations in a year or 1 exacerbation leading to hospitalisation.

**Figure 1:**
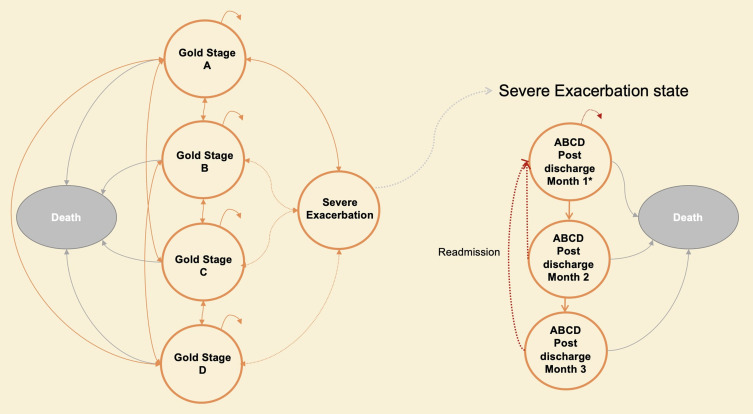
Model Schematic.

**Notes**: *Hospital admission and readmission all carry unique probability of death on admission, not shown in the diagram to avoid further cluttering.  Possible stage Deterioration after exacerbation.

From the second year onwards patients moved through the model as shown in Figure 1. A previous COPD model developed by the author team^16^ was updated to reflect recent COPD classifications.^17^ Patients transitioned between GOLD stages reflecting prognosis within the BLISS cohort study^18^ (described in Appendix 4), where patients were categorised into GOLD stages A-D at 12-month and 24-month follow-up.

When the patient had a severe hospitalised exacerbation, they occupied 3 post-discharge tunnel states for 3-months reflecting their reduced quality of life. As GOLD stage is partly based upon admission history patients in COPD A and B logically moved to C and D after an admission. A transition matrix reflecting movements between each of the stages over the second year of the study, is shown in Table S6 and Appendix 3.

##### Modelled Treatment Effect

There were four independent treatment effects relating to COPDPredict™ in the model. The first effect was shown in Table 3, eg, the benefit from improved starting states. Secondly, a lower rate of severe exacerbations was detected in the trial on COPDPredict™ meaning fewer patients will transition from Stages A and C to Stages B and D on the intervention. The usual care transition matrix shown in Table S6 was adjusted to reflect expert advice that the anticipated likely future benefit of COPDPredict™ upon exacerbations would continue but decline by 50% each year until the end of Year 5 (shown in Table 4). New transition probabilities for COPDPredict™ patients were calculated and are shown for year 2–5 in Table 4.

**Table 4:** Monthly Transition Probabilities and Exacerbation Rates for COPDPredict™ Model Arm, yr 2–5 with PSA Distribution Parameters

**Transition Matrix**				
**GOLD stage^c^ 1yr / 2yrs**	**A Proportion (alpha, beta)**	**B Proportion (alpha, beta)**	**C Proportion (alpha, beta)**	**D Proportion (alpha, beta)**
A	#	0.008 (2, 282)	0.011 (3, 281)	0.003 (1,283)
B	0.013 (3, 225)^a^	#	0.002 (1, 227)	0.018 (4, 224)
C	0.030 (3, 93)	0.054 (0.5, 95.5)	#	0.006 (0.5, 95.5)^a^
D	0.003 (1, 253)	0.015 (4, 251)	0.004 (1, 253)^a^	#
**Monthly exacerbation rate by GOLD Stage**				
**COPD stage^c^**	**Mean monthly exacerbation rate**		**Alpha**	**Beta**
A	0.002		44.99	20,743.01
B	0.007		85.88	12,839.12
C	0.005		18.82	3466.174
D	0.015		75.57	5057.422
**COPDPredict™ treatment effect**				
**Relative risk of exacerbation on COPDPredict™**	**Mean^b^**		**Standard error^b^**	
Yr 2	0.91		0.604	
Yr 3	0.955		0.622	
Yr 4	0.978		0.636	
Yr 5	0.989		0.643	

**Notes**: # Indicates patients not moving states each cycle remain in the same state. ^a^Green shading shows beneficial transitions for COPDPredictTM relative to usual care (alphas and betas rounded to 1sf). ^b^Lognormal distribution used to sample relative risks in probabilistic sensitivity analysis. ^c^GOLD A (low risk, fewer symptoms), B (low risk, more symptoms), C (high risk, fewer symptoms), and D (high risk, more symptoms). Fewer symptoms reflects COPD Assessment Test (CAT) score <18, more symptoms CAT score ≥18. High risk reflects patients with ≥2 exacerbations in a year or 1 exacerbation leading to hospitalisation.

Third, for patients in states B and D, defined by a hospitalised exacerbation, fewer exacerbations would occur on the intervention. Mean exacerbations per stage were taken from BLISS^18^ (Table 4) and multiplied by the relative risks to obtain the exacerbation rate on the intervention.

Finally, those who had a hospitalised exacerbation had 1.278 (0.914) fewer bed days per exacerbation when receiving COPDPredict™.

#### Utility Values

During the first year of the model, utility values directly reflected those from trial data (Table S4). From 12 months onwards, utility values for each COPD stage for both comparators reflected mean utility values for patients at 12-month follow-up in the BLISS cohort (shown in Table 5). Disutility for hospitalised exacerbations were also obtained from the BLISS data.^18^

**Table 5:** Year 2–5 Utility Values and Costs for GOLD Stages and Hospitalised Exacerbations with PSA Distribution Parameters

**Utility Values by Health State**			
**Health state**	**EQ-5D Coefficients^a^**	**Alpha**	**Beta**
GOLD A	0.83	65.56	13.05
GOLD B	0.64	145.37	83.56
GOLD C	0.82	70.86	15.47
GOLD D	0.59	164.33	115.29
Hospitalised exacerbation (disutility for 3 months)	−0.06	376.50	6048.44
**Costs by Health State**			
**COPD GOLD stage^b,c^ monthly costs**	**Cost mean (SE)**	**Gamma κ**	**Gamma *θ***
A	Stage B costs minus 33.10 (48.41)	0.47	70.80
B	£48.51 (22.34)	4.75	10.29
C	Stage D costs minus 33.10 (48.41)	0.47	70.80
D	£75.35 (18.56)	16.43	4.57
Hospitalised exacerbation^b^	£2258.33 (313.26)	Normally sampled	
Cost of hospitalisation bed day^b^	£260.71 (44.12)		
COPDPredict™ cost^b,+^	£39.90 (20.00)	3.98	10.03

**Notes**: ^a^Calculated by OLS regression analysis controlling for age and gender and GOLD stage. ^b^Gamma distributions were used to sample cost parameters to prevent negative cost sampling. ^C^ GOLD A (low risk, fewer symptoms), B (low risk, more symptoms), C (high risk, fewer symptoms), and D (high risk, more symptoms). Fewer symptoms reflects COPD Assessment Test (CAT) score <18, more symptoms CAT score ≥18. High risk reflects patients with ≥2 exacerbations in a year or 1 exacerbation leading to hospitalisation. ^+^Cost of the intervention in subsequent years plus the cost of monthly home visits. 50% standard error assumed for PSA.

#### Costs

During the first year of the model, the modelled data directly reflected the imputed costs obtained from the trial (Table 2).

##### Costs Years 2–5

For the modelled analysis, those on the COPDPredict™ intervention incurred costs associated with the intervention (Table 1), in addition to the mean costs associated with membership of each COPD stage. Stage membership costs were estimated independent of hospitalised exacerbation costs but included non-hospitalised exacerbation costs (Table 5). Costs were taken from the trial data because of the more comprehensive level of cost included compared with the BLISS cohort data.^18^ GLM Regression techniques were used to estimate costs associated with stages A and C by controlling for costs associated with exacerbations and subtracting them from states B and D. Hospitalised exacerbations were estimated using the prices shown in Appendix 1, with COPDPredict™ hospitalised exacerbation costs reduced to reflect fewer bed days.

#### Key Model Assumptions

- Differences in hospital bed days identified for hospitalised exacerbations were sustained over 5 years.
- The differences in transitions and exacerbations per COPD stage were reduced by 50% per cycle.
- Transition and utility values from the BLISS cohort were appropriate for current stage D patients.
- Transitions calculated from the BLISS data from 12 to 24 months were appropriate for use to extrapolate 3–5 years.
- Trial data can be used to price costs of both treatments beyond 12 months.

#### Sensitivity Analysis

Deterministic analysis explored the impact of uncertainty in key model parameters, as well as methodological and structural decisions, on model outputs. These include the following alternative scenarios;

- Intervention costs
- Reduction in bed days
- Utility gains on the intervention
- Overall severe exacerbation rates
- Effect of intervention on severe exacerbations
- Starting states

Recognising the proposed updates to the GOLD categorisation of COPD severity, we included a sensitivity analysis reflecting the new GOLD classifications where A and B groups are unchanged but C and D are merged into a single group termed “E” which is >2 moderate exacerbations or >1 hospitalised exacerbation (detailed methods in Appendix 5).

For Probabilistic sensitivity analysis (PSA), distributions were constructed for each model parameter, and PSA was performed using 10,000 replications with sampling from the distributions. Distributions are stated in the model parameter tables, but generally beta distributions were used for probabilities and utility values, normal and gamma distributions for costs, and log-normal distributions for relative risks.

Cost-effectiveness planes were plotted to show the distribution of incremental costs and QALYs, and CEACs were constructed to reflect the intervention cost-effectiveness probability at different WTP per QALY thresholds. The Expected Value of perfect information (EVPI) analysis was undertaken to explore the value of further research, and the Expected Value of Perfect Parameter Information (EVPPI) to identify where that research should be targeted.

## Results

### Trial Based Analysis

#### Base Case Analysis Imputed Over 6-Months

There were considerable improvements in EQ-5D over the first six months on the intervention (Table 2). Accordingly, on average, COPDPredict™ was associated with 0.036 more QALYs over 6-months. However, COPDPredict™ was also associated with higher NHS costs, particularly related to the cost of the intervention and home visits, while the costs associated with hospitalisations were lower.

An economic analysis was not performed from a societal perspective because few patients were employed (n=15 at 6-months), skewing related to outliers (one patient had 61 days of work absence on usual care), and missingness (>50% at 12-months). Nonetheless it is worth noting that private healthcare and patient-incurred costs were £22.65 lower for COPDPredict™. Appendix 2 presents selected work-related outcomes.

#### Cost Utility Analysis

When adjusting for baseline randomisation variables and applying non-parametric bootstrapping of costs and QALYs, COPDPredict™ was associated with £238.16 additional NHS costs with a QALY gain of 0.020 (Table 6).

**Table 6:** Imputed Descriptive and Bootstrapped Adjusted Incremental Health Outcomes and Costs Over 6 Months. Values are Mean (SE) Scores Unless Stated Otherwise

Adjusted Bootstrap COPD Predict Treatment Effect 6-Month^a,b,c^				
	Mean Inc. QALY	Mean Inc. NHS costs (£)	ICER (£/QALY)	Probability Cost-Effective^d^
COPDPredict™	+0.020 (0.012)	+238.16 (106.42)	£11,669	65%

**Notes**: ^a^Chained imputation adjusted for disease severity, gender, age, site and primary outcome. Primary outcome (hospitalisations) included in the imputation for costs. Imputation for 13 patients, (n=9 COPDPredict™, n=4 usual care). 10 patients returned zero questionnaires post baseline and were excluded from the imputation. ^b^Bootstraps adjusted for baseline EQ-5D, disease severity, gender, age and site. ^c^Deaths excluded from the analysis. ^d^Probability Cost-Effective at a £20,000/QALY willingness to pay threshold.

The intervention was 65% likely to be cost-effective with an ICER of £11,669/QALY.

**Figure 2:**
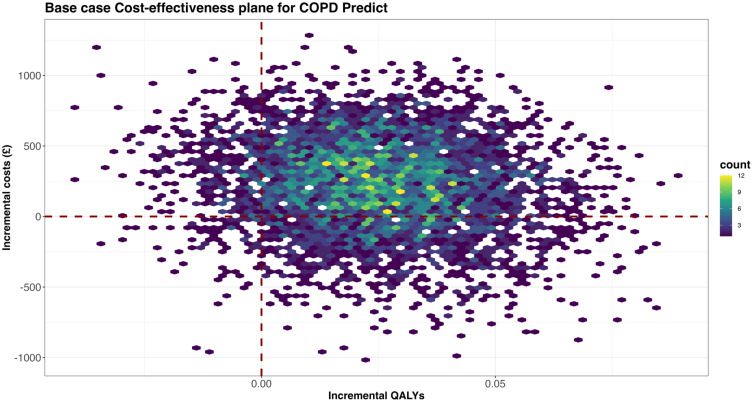
Adjusted bootstrapped imputed cost utility plane comparing COPDPredict™ with usual care at 6-months.

Figure 2 shows the distribution of bootstrapped costs and effects, with most samples showing an incremental gain in QALY, suggesting that the intervention is very likely to be effective.

**Figure 3:**
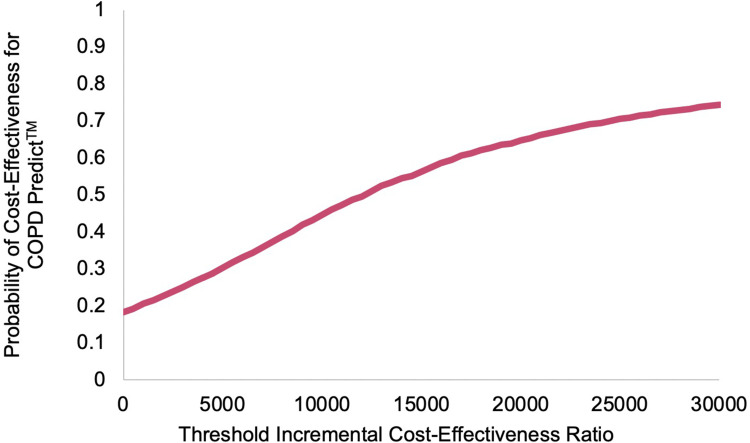
Adjusted bootstrapped imputed cost effectiveness acceptability curve comparing COPDPredict™ with usual care at 6-months.

When plotted on the cost-effectiveness acceptability curve, the intervention rises to 74% likelihood of cost-effectiveness at the upper bound of the NICE willingness-to-pay threshold of £30,000/QALY (Figure 3).

#### Cost Effectiveness Analysis

The cost-effectiveness analysis in Table 7 shows that COPDPredict™ was associated with a reduction in hospitalisations of 0.018 over 6-months, with bootstrapped adjusted analysis showing a mean reduction of 0.016 in hospitalisations.

**Table 7:** Imputed Incremental Hospitalisations and Costs Over 6 Months. Results are Adjusted for Baseline Randomisation Variables

**Mean costs, QALYs and ICER imputed^a,b^**				
	***Unadjusted 6-months***			
**Strategy**	**N**	**Mean Hospitalisations**		**Mean NHS costs**
COPDPredict™	39	0.128 (0.066)		£1059.69 (155.08)
Usual care	41	0.146 (0.066)		£856.68 (200.63)
Unadjusted difference	80	−0.018 (0.093)		+£203.01 (255.36)
***Adjusted bootstrap COPD Predict Treatment effect 6-month^a,b,c^***				
	**Mean Inc. Hospitalisations**	**Mean Inc. NHS costs (£)**	**ICER (£/Hospitalisation averted)**	**Probability reduction in hospitalisations**
COPDPredict™	+0.016 (0.102)	+198.30 (287.85)	£12,394	57%

**Notes**: ^a^Chained imputation adjusted for disease severity, gender, age, site. Imputation for 5 patients, (n=4 COPDPredict™, n=1 usual care). 10 patients returned zero questionnaires post baseline and were excluded from the imputation. ^b^Deaths excluded from the analysis. ^c^Bootstraps adjusted for disease severity, gender, age and site.

The ICER estimates a cost of £12,394 to avert a hospitalisation with a 57% probability of a reduction in hospitalisations over 6-months.

#### Sensitivity Analysis

Cost-utility results were robust to methodological approach, as Table 8 shows, the complete case results yield similar results at 6-months, albeit slightly higher probability of cost-effectiveness at 68% rising to 78% at £30,000/QALY.

**Table 8:** Regression Adjusted and Bootstrapped Incremental Health Outcomes and Costs Over 6- and 12-Months. Complete Case Analyses. Values are Mean (SE) Scores Unless Stated Otherwise

**Adjusted bootstrap COPD Predict Treatment effect 6-months^a,b,c^**				
	**Mean Inc. QALY**	**Mean Inc. NHS costs (£)**	**ICER (£/QALY)**	**Probability Cost-Effective^d^**
COPDPredict™	+0.025	+224.61	8984	68%
**Adjusted bootstrapped COPD Predict Treatment effect 12-months^b,c,e^**				
COPDPredict™	+0.073 (0.052)	+246.31 (928.91)	3374	82%

**Notes**: ^a^Complete case at 6-months reflects all patients with completed responses to all EQ-5D and healthcare resource questionnaires at 3 and 6 months, n=30 for COPDPredict™ and n=37 for usual care. ^b^Adjusted for baseline EQ-5D, disease severity, gender, age and site. ^c^Deaths excluded from the analysis. ^d^Probability Cost-Effective at a £20,000/QALY willingness to pay threshold. ^e^Complete case at 12-months reflects all patients with completed responses to all EQ-5D and healthcare resource questionnaires at 3, 6, 9 and 12 months months n=18 for COPDPredict™, n=25 for usual care.

A complete case analysis over 12-months was more speculative, as only 43 patients returned all relevant data points. COPDPredict™ was still associated with a considerable QALY gain at a modest additional NHS cost when bootstrapping and adjustment are considered.

When imputing all surviving patients over 12-months Table 9 shows that the incremental costs of COPDPredict™ may be higher (+£526.38) than the complete case analysis suggests, with an accompanying smaller QALY gain (+0.019), resulting in an ICER above £20,000/QALY and a likely cost-effectiveness probability of 43%, increasing to 52% at £30,000/QALY.

**Table 9:** Regression Adjusted and Bootstrapped Imputed Incremental Health Outcomes and Costs Over 12 Months. Values are Mean (SE) Scores Unless Stated Otherwise. N=78

	Adjusted Bootstrap COPD Predict Treatment effect 12-Months^+ab^			
	Mean Inc. QALY	Mean Inc. NHS Costs (£)	ICER (£/QALY)	Probability Cost-Effective^c^
COPDPredict™	+0.019 (0.034)	+526.38 (477.71)	£27,133/QALY	43%

**Notes**: ^a^Chained imputation adjusted for disease severity, gender, age, site. 10 patients returned zero questionnaires post baseline and were excluded from the imputation. N=19 patients were imputed for COPDPredict™ and n=16 for usual care. ^b^Adjusted for disease severity, gender, age and site. QALYs adjusted for baseline EQ-5D. ^c^Probability Cost-Effective at a £20,000/QALY willingness to pay threshold. ^+^Deaths within 12-months excluded from the analysis.

### Model-Based Analysis

#### Base Case

The base case model results are shown in Table 10.

**Table 10:** Base Case Model Estimated Incremental Health Outcomes and Costs Over 5 years

Mean NHS Costs, QALYs and ICER Base Case Model		
Strategy	Mean QALYs	Mean NHS Costs
COPDPredict™ ^a,b^	2.614	£6239
Usual care	2.482	£4718
Incremental difference	+0.132	+£1521
ICER (£/QALY)		£11,862
Probability cost-effective^c^		66%

**Notes**: ^a^1.28 day reduction in length of stay for exacerbation. ^b^12% (yr2), 6% (yr3), 3% (yr4), and 1.5% (yr5) reduction in exacerbations on COPDPredict™ in stages B-D. ^c^Probability Cost-Effective at a £20,000/QALY willingness to pay threshold.

**Figure 4:**
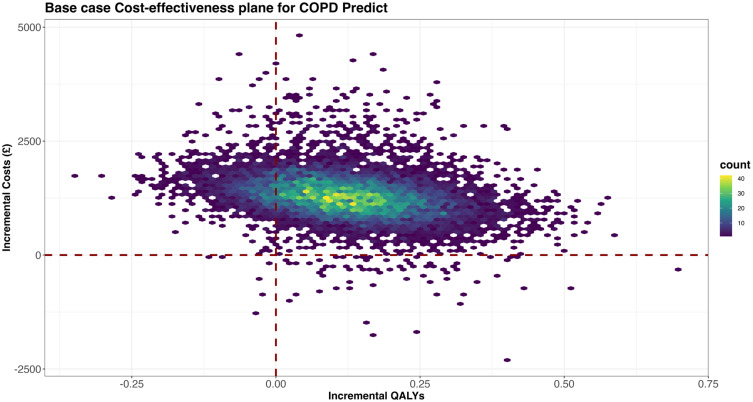
Base case model cost utility plane comparing COPDPredict™ with usual care over 5 years.

The intervention is likely to be cost-effective, with an ICER of £11,862/QALY. The PSA results in Figure 4 show that it is highly likely that the intervention provides incremental QALY gains, albeit at additional costs.

**Figure 5:**
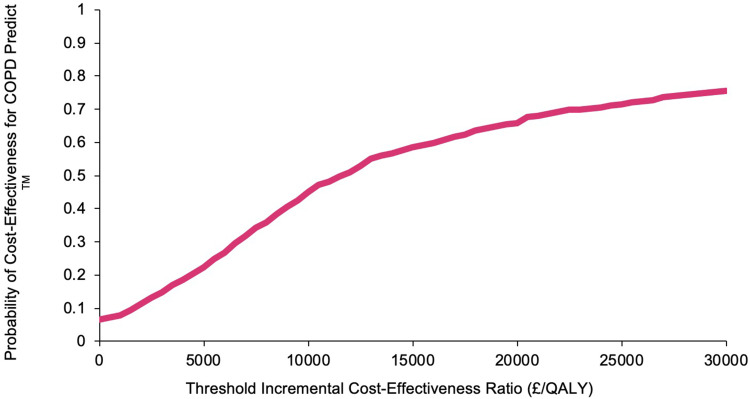
Base case modelled cost effectiveness acceptability curve comparing COPDPredict™ with usual care over 5 years.

The CEAC in Figure 5 shows a 68% probability of cost-effectiveness at £20,000 per QALY, which increases to 76% at £30,000 willingness to pay.

#### Deterministic and Scenario Analysis

The sensitivity analysis showed that the results were very sensitive to the costs and outcomes of the intervention (Appendix 6 and Table S14). If intervention costs were greater than those in the trial, then the intervention would be less cost-effective, and vice versa. If the reduction in bed days was not sustained beyond the first year, cost-effectiveness probability would decrease to 62%.

The base-case model did not include an intervention utility benefit, independent of reducing exacerbations. Cost-effectiveness would be much higher (rising to 92%), with an annual 0.04 improvement of EQ-5D score independent of the exacerbation effect.

As few patients experienced hospitalised exacerbations (<20%, even in COPD stage D), the model is robust to differing assumptions regarding interventions that reduce hospitalised exacerbations. However, the baseline severe exacerbation rates in the trial (and the BLISS cohort^13^) were much lower than those estimated by a recent analysis of COPD patients using CPRD and HES data with 44,000 patients.^19^ Accordingly, a sensitivity analysis was performed using higher estimates, with a significant improvement in the likelihood of the cost-effectiveness of COPDPredict™. Starting states are far more influential on cost-effectiveness if they are assumed to be equal at 12-months; then, cost-effectiveness falls to an ICER above the £20,000 NICE threshold.

When using a model structure with the GOLD ACE health states (Appendix 5 and Table S13) the ICER for the intervention falls just above the threshold (£21,689/QALY) with the PSA showing 48% probability of cost-effectiveness at the £20,000 NICE threshold, rising to 61% probability at £30,000/QALY (Figure S2).

#### Expected Value of Perfect Information

**Figure 6:**
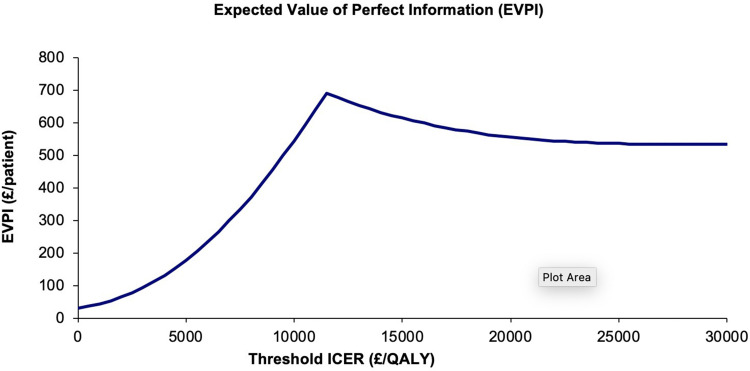
Expected value of perfect information curve.

Unsurprisingly, given the small sample sizes and uncertainty, there is expected to be considerable value in further information; at a willingness to pay of £20,000/QALY, the EVPI is £556 per patient (Figure 6).

Appendix 7 and Table S15 describe the methods used to obtain the estimated population expected to benefit from the intervention, 626,129, which is used to multiply per-patient costs. Table S16 in Appendix 6 shows that there is significant value in future research when the population size is considered, future research should target stage transitions and mortality rates, particularly for COPDPredict™.

## Discussion

### Main Findings and Interpretation

The results of both trial- and model-based economic analyses provide evidence that the COPDPredict™ system is cost-effective for COPD stage D patients in the UK. The strong likelihood of cost-effectiveness is driven primarily by an improvement in EQ-5D scores seen in the trial, as well as a reduction in hospitalised exacerbations which influences the long-term trajectory of patients in the model-based analysis.

Model-based results suggest that the intervention is likely to continue to be cost-effective when used over the longer-term. The trial-based and model-based analyses produced similar ICERs despite the additional period to benefit within the model. This is because the relative risk reduction in exacerbations is assumed to decline in magnitude over five years in the model, and the full cost of the licence fee for the intervention is also incurred over five years. Note, long-term cost-effectiveness modelling is predicated upon some continued reduction in exacerbations and bed days experienced from the intervention, improved COPD Gold state, and lower costs, which are currently based on small sample sizes in the trial and are therefore uncertain.

Sensitivity analysis can help understand the impact of these assumptions upon intervention cost-effectiveness. Whilst the model is somewhat robust to alternative assumptions regarding the period of benefit and even choice of model structure, the results are highly sensitive to small changes in costs. For example, if costs were reduced, perhaps by tailoring home-visit routines more efficiently to target patients most in need, the cost-effectiveness of the intervention would improve considerably. Equally, it is important to continue to look for ways to minimise costs to ensure that increases in costs do not render the intervention too expensive.

The base-case model is conservative in some respects and possibly underestimates the benefit of the intervention upon quality of life, which the trial-based analyses suggest could go beyond merely reducing exacerbations. If even minor improvements in quality of life were sustained during the model, this would lead to improved cost-effectiveness of the intervention. Moreover, the trial suggested low rates of hospitalised exacerbations across this population; however, if hospitalised exacerbation rates were higher, the cost-effectiveness of the intervention would improve significantly. This is a logical implication, as a relative risk reduction has a greater effect on the higher the base number it impacts. Therefore, another logical implication is that the intervention may be more effective in more frequently exacerbating patients.

Within the trial-based analysis, the imputed 12-month results suggest that there may be some uncertainty in the likely cost-effectiveness of the intervention, although it should be noted that imputing over 50% of the observations for the intervention should be considered highly speculative.

Whilst a novel methodology applying the new proposed GOLD ACE health states was presented, caution is proposed over the use of such classifications in long-term modelling of COPD patients. In economic modelling any attempt to merge health states can potentially reduce granularity and therefore the sensitivity of models to potential improvements in longer-term health prognosis. In this case GOLD C is superior to GOLD B and D (higher quality of life and lower costs), and therefore merging C and D into GOLD E dilutes the modelled benefit of COPDPredict™ over time. In this model this occurs in two separate ways; firstly, in the ABCD model 6% of patients initially move into GOLD C on COPDPredict™ and 0% on usual care, a benefit which is negated in the ACE model. Secondly, throughout the ABCD model COPDPredict™ has the effect of increasing GOLD B membership at the expense of GOLD D, As GOLD E has both a higher utility value and lower cost than GOLD D, this benefit is diluted for both costs and quality of life. We recommend health economists give consideration to the likely impact of the modelled intervention upon condition-specific disease processes before selecting an appropriate structure.

### Implications for Clinical Practice

The results of these analyses could carry significant implications for clinical practice. Clinicians should recognise the potential that integration of app-based self-management tools into routine care could provide for the enhancement of patient outcomes by empowering patients with real-time support as well as education. Additionally, clinicians should note the potential benefits associated with reduced hospital admissions, such as freeing beds for the treatment of other patients. Successfully integrating COPDPredict™ does necessitate training for both healthcare professionals and patients to ensure effective utilisation and maximise the potential benefits of app-based self-management programs within the clinical setting. This necessitates consultant oversight of the dashboard (or training others to a sufficient level to do so) as well as the dashboard requiring a 7-day service for prompt response to alerts.

### Strengths and Limitations

The strengths of our trial-based analysis lie in the presentation of both trial- and model-based analyses of the intervention, providing a broader understanding of the potential benefits and costs of the intervention as well as the degree of uncertainty and points of sensitivity.

The model itself represents a novel methodology in that it applies recent GOLD classifications to produce an original model which reflects recent understanding of the disease with progression through a model reflecting exacerbation frequency, which is critically important for this population. Analysing the Birmingham COPD cohort study allowed the generation of estimates on transitions between stages of ABCD for over 1000 patients, which were provided as transition probabilities and could be used to model future COPD ABCD models instead of the older models based on COPD 1–4. We also present the same innovation for newly proposed COPD ACE health states.

The model relies on assumptions regarding long-term treatment impacts derived from small sample sizes. However, decision-modelling methods have been used to estimate the impact of, and quantify the value of a reduction in, uncertainty. Broad deterministic sensitivity analyses were performed to account for the uncertainty in structural and methodological decisions. Probabilistic sensitivity estimates the probability of cost-effectiveness, given the joint uncertainty in the input parameters, and accounts for the small sample sizes used in the model. Expected Value of Information was used to quantify the benefit of a reduction in uncertainty and guide further research.

Clearly, small sample sizes impact the degree of confidence in the results obtained from both trial-based and model-based analyses. Further caution is warranted, given the results of the imputed analysis over 12-months suggest the ICER is close to the £30,000 WTP threshold for QALY. The intention-to-treat analyses also contain a high degree of imputation, reflective of the small sample sizes returning complete data in the trial (n=67 for 6-months and n=43 for 12-months) dictating that the results should be treated with caution.

The generalisability of the results should be considered within the context of the trial population (see Appendix 8); almost exclusively COPD D patients, and only patients with COPD D entered the model cohort. In other words, similar results may not be obtained for the other groups.

### Future Research

Although early evidence is promising, more evidence on the long-term impact of COPDPredict™ based on a large cohort of patients is required to inform economic analyses. The Expected Value of Perfect Parameter information shows that there is significant value in further research regarding long-term transitions when using the intervention, as well as evidence from larger sample sizes regarding mortality. Meanwhile, deterministic sensitivity analyses demonstrate the need to obtain more information regarding the assumptions within the model, eg long-term reduction in bed days, maintenance of improved starting states, and long-term costs. The model-based analysis only runs for 5-years given the period the intervention is likely to impact cost-effectiveness, and any further benefit is not modelled here.

Currently, most of the patients in the trial had COPD D; hence, little is known about the effectiveness of the trial in other COPD severity grades. Future trials should look to improve the knowledge about the behaviour of the intervention in this patient group, which would also facilitate modelling of this group.

## Conclusion

COPDPredict™ appears to be cost-effective for patients with COPD over the short- and long-term, up to a 5-year time horizon. Cost-effectiveness is potentially greater in more frequent hospitalised exacerbators. Uncertainty remains around the cost-effectiveness of the intervention, given the small sample sizes and the use of assumptions; further work is required.

## Data Availability

All data relevant to the modelling study are included in the article or uploaded as supplementary information. We do not intend to share primary individual participant data for the trial-based analysis.

## Predict and Prevent Trial Registration

Full title “The use of a personalised early warning decision support system with novel saliva bio-profiling to predict and prevent acute exacerbations of Chronic Obstructive Pulmonary Disease - ‘Predict & Prevent AECOPD’” Clinicialtrials.gov Identifier NCT04136418.
